# Crystal structure of zwitterionic 3,3′-[1,1′-(butane-1,4-di­yl)bis­(1*H*-imidazol-3-ium-3,1-di­yl)]bis­(propane-1-sulfonate) dihydrate

**DOI:** 10.1107/S2056989020009779

**Published:** 2020-07-24

**Authors:** Antal Udvardy, Sourav De, Tamás Gyula Gál, Gábor Papp, Csilla Enikő Czégéni, Ferenc Joó

**Affiliations:** a University of Debrecen, Department of Physical Chemistry, PO Box 400, Debrecen, H-4002, Hungary; b University of Debrecen, Doctoral School of Chemistry, PO Box 400, Debrecen, H-4002, Hungary; c MTA-DE Redox and Homogeneous Catalytic Reaction Mechanisms Research Group, PO Box 400, Debrecen, H-4002, Hungary

**Keywords:** crystal structure, di-*N*-heterocyclic carbene precursor, hydrogen bonds, water solubility

## Abstract

The crystal structure of the title compound, C_16_H_26_N_4_O_6_S_2_·2H_2_O, a water-soluble di-*N*-heterocyclic carbene ligand precursor was determined using a single crystal grown by the slow cooling of a hot *N*,*N*-di­methyl­formamide solution of the compound. The dihydrate crystallizes in the monoclinic space group *P*2_1_/*c*, with half of the zwitterionic mol­ecule and one water mol­ecule of crystallization in the asymmetric unit. The remaining part of the mol­ecule is completed by inversion symmetry. In the mol­ecule, the imidazole ring planes are parallel with a plane-to-plane distance of 2.741 (2) Å. The supra­molecular network is consolidated by hydrogen bonds of medium strength between the zwitterionic mol­ecules and the water mol­ecules of crystallization, as well as by π–π stacking inter­actions between the imidazole rings of neighbouring mol­ecules and C—H⋯O hydrogen-bonding inter­actions.

## Abstract 2

A known *N*-heterocyclic carbene precursor, zwitterionic 3,3′-[1,1′-(butane-1,4-di­yl)bis­(1*H*-imidazol-3-ium-3,1-di­yl)]bis­(propane-1-sulfonate) crystallized as its dihydrate and was characterized by single-crystal X-ray diffraction, revealing point group symmetry 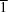 for the zwitterionic mol­ecule.

## Chemical context   

Imidazolium salt-based ionic liquids are versatile because of their unique properties and their use as green solvents, replacing volatile or toxic organic solvents (De *et al.*, 2019 ▸). Moreover, they are very often used as reaction media, or – the water-immiscible ones – for extraction. X-ray crystallographic studies of several crystalline imidazolium salts have been described. However, examples of zwitterionic imidazolium salts are limited in the literature, and only a few examples of zwitterionic imidazolium sulfonates, with their crystal structures determined, have been reported to date. The introduction of hydro­philic substituents (*e.g*. sulfonate groups) made possible the synthesis of water-soluble metal complexes, and subsequently, a range of catalytic applications (Kohmoto *et al.*, 2012 ▸).
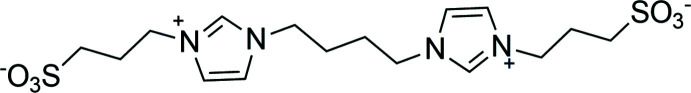


**Figure 1:**
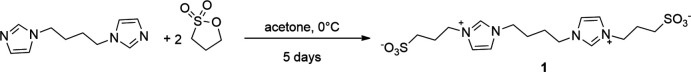
Synthesis scheme of **1**.

Here we report the crystal structure of the title compound, 3,3′-[1,1′-(butane-1,4-di­yl)bis­(1*H*-imidazol-3-ium-3,1-di­yl)]bis­(propane-1-sulfonate) (**1**; Fig. 1 ▸), which crystallizes as a dihydrate (**1**·2H_2_O). To the best of our knowledge, this is the first crystal structure determination of an alkyl­ene-bridged di-imidazolium salt with ω-propyl­sulfonate wingtips. Compound **1** is known from the literature (Liu *et al.*, 2013 ▸; Xu *et al.*, 2012 ▸; Zeng *et al.*, 2013 ▸) and was prepared according to the method described by Papini *et al.* (2009 ▸), utilizing the reaction between 1,1′-(butane-1,4-di­yl)di-1*H*-imidazole and 1,3-propane­sultone (Fig. 1 ▸).

## Structural commentary   

The di-*N*-heterocyclic carbene precursor **1** crystallizes as a dihydrate, with one half of the mol­ecule and one water mol­ecule of crystallization being present in the asymmetric unit. The other half of the mol­ecule is generated by the application of inversion symmetry (symmentry operation: 1 − *x*, −*y*, −*z*). No molecules of the solvent, DMF, from which the crystals were obtained, are built into the lattice.

**Figure 2:**
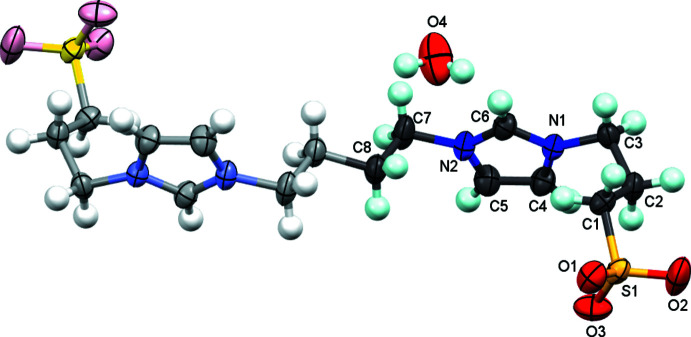
The mol­ecular structure of **1** showing the atom labelling and displacement ellipsoids drawn at the 50% probability level. The asymmetric unit of **1** is given in darker colours, and the symmetry-generated part (symmetry code: 1 − *x*, −*y*, −*z*) of the mol­ecule is given in lighter colours. The water mol­ecule is also shown.

**Figure 3:**
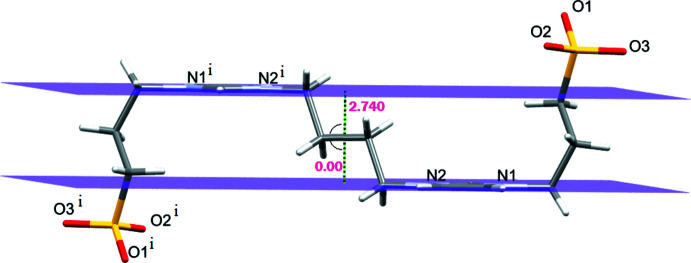
The imidazole ring planes in **1** displayed as capped sticks. The water mol­ecules of crystallization are omitted for clarity [Symmetry code: (i) 1 − *x*, −*y*, −*z*].

The zwitterionic mol­ecule of **1** (Fig. 2 ▸) is composed of two imidazolium propane sulfonate fragments, which are linked by a butyl­ene bridge. The N1—C6 and N2—C6 bond lengths are 1.327 (3) Å and 1.320 (4) Å, and the N1—C1—N2 angle is 108.9 (2)°. The length of the C2—C3 bond of 1.342 (4) Å indicates that these carbon atoms are *sp* ^2^ hybridized. The sulfonate moiety is rigid, with characteristic bond lengths of S1—C1 = 1.773 (3) Å, S1—O1 = 1.446 (2) Å, S1—O2 = 1.450 (2) Å, S1—O3 = 1.453 (2) Å, and angles O1—S1—O2 = 112.16 (14)°, O1—S1—O3 = 111.80 (14)°, and O2—S1—O3 = 112.80 (15)°. As a result of the point group symmetry 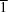 of the mol­ecule, the imidazole planes are parallel and have a distance of 2.741 (2) Å (Fig. 3 ▸) from each other.

## Supra­molecular features   

**Figure 4:**
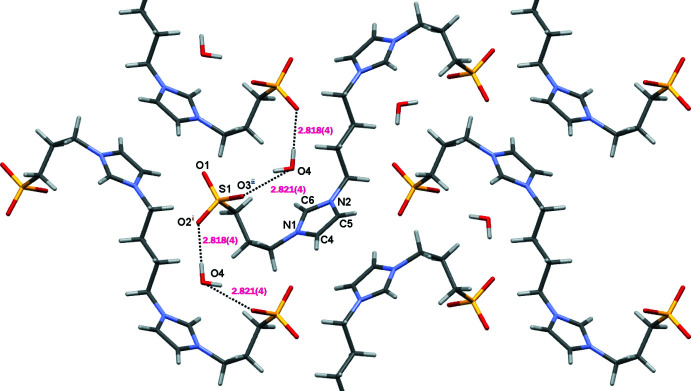
A partial packing diagram of **1**, showing the formation of ribbons through O—H⋯O hydrogen bonds. [Symmetry codes: (i) *x* + 1, −*y* + 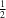, *z* − 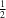; (ii) *x* + 1, *y*, *z*].

**Figure 5:**
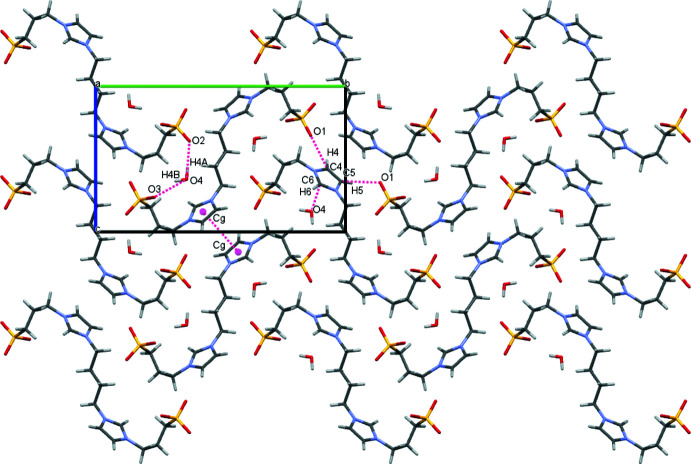
Packing diagram of **1** viewed along the *a* axis, showing selected hydrogen bonds (including weak C—H⋯O inter­actions) and π–π stacking inter­actions (*Cg* is the centroid of the: N1–N6–N2–C4–C5 ring).

The water mol­ecules bridge adjacent zwitterionic mol­ecules through hydrogen bonds of medium strength with sulfonate O atoms as acceptor groups into ribbons aligned parallel to [001] (Table 1 ▸, Fig. 4 ▸). π–π stacking inter­actions involving the imidazole rings of neighbouring mol­ecules (symmetry operation 2 − *x*, 1 − *y*, 1 − *z*; centroid-to-centroid distance of 3.9541 (17) Å, slippage 1.622 Å, Fig. 5 ▸) lead to the formation of supra­molecular layers extending parallel to (100). Additional weak C—H⋯O hydrogen bonds (Table 1 ▸, Fig. 5 ▸) consolidate the three-dimensional network structure.

## Database survey   

**Figure 6:**
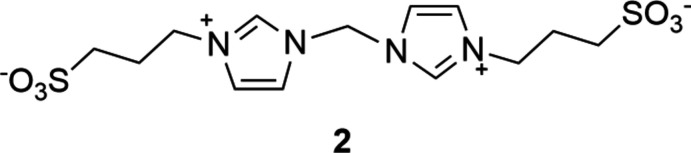
Structural formula of 3,3′-[1,1′-methyl­enebis(1*H*-imidazole-3-ium-3,1-di­yl)]bis­(propane-1-sulfonate (**2**).

A search of the Cambridge Structural Database (CSD Version 5.41, May 2020; Groom *et al.*, 2016 ▸) revealed no similar crystal structures of di-*N*-heterocyclic carbene ligand precursor mol­ecules where two *N*-ω-sulfonato­propyl-imidazolium units are connected through an α,ω-alkyl­ene bridge. Crystals of similar sulfoalkyl-imidazolium di-NHC precursors containing aromatic linkers have been grown by Kohmoto *et al.* (2012 ▸). Crystal structures of gold(III) (refcode: KOGGUK; Hung *et al.*, 2014 ▸) and palladium(II) (Asensio *et al.*, 2017 ▸) metal complexes with similar ligands with a methyl­ene bridge (Fig. 6 ▸, **2**) were also reported.

The Pd complexes [PdCl_2_(**2**)], refcode: YAVROF, and [Pd(**2**)_2_], refcode: YAVXAX, crystallize in space group type *P* 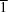. In all other above-mentioned cases, the space-group type is *P*2_1_/*c*.

## Synthesis and crystallization   

Compound **1** was synthesized according to the method of Papini *et al.* (2009 ▸). 4.4 mmol of 1,3-propanesultone were slowly added to a solution of 2 mmol of 1,1′-(butane-1,4-di­yl)di-1*H*-imidazole in 30 ml of acetone at 273 K. Then the mixture was left to warm to room temperature and stirred for 5 d. The solvent was evaporated and the resulting white solid was recrystallized from methanol affording **1** as a white powder (yield: 617 mg, 71%). Analytical data: ^13^C{^1^H}-NMR (90 MHz, D_2_O, 298 K) δ [ppm], 135.6, 122.5, 48.8, 47.8, 47.2, 26.2, 25.0; ESI–MS (CH_3_OH, positive mode), *m*/*z* observed 435.1359, calculated value for C_16_H_27_N_4_O_6_S_2_, ([*M* - H]^+^): 435.1367). For recrystallization, **1** was suspended in DMF and heated to approximately 373 K, then filtered and left overnight to slowly cool down to room temperature. Single crystals, suitable for X-ray analysis, were obtained as colourless prisms after storing the solution in open glass vials in a refrigerator at 278 K. A possible source of water is the employed DMF, which is hygroscopic and easily adsorbs water from a humid atmos­phere. The same type of prismatic crystals were also grown from hot water, revealing a very similar unit cell. However, these crystals were of poor quality, and the best *R*_int_ value was very high, 0.19.

## Refinement   

Crystal data, and details of data collection and structure refinement are summarized in Table 2 ▸. Hydrogen atoms of the zwitterionic mol­ecules were placed at idealized positions and refined using a riding model. The positions of hydrogen atoms of the water mol­ecule were discernible in a difference-Fourier map. They were refined with a fixed bond length of 0.85 Å and *U*_iso_(H) = 1.5*U*_eq_(O).

## Supplementary Material

Crystal structure: contains datablock(s) I. DOI: 10.1107/S2056989020009779/wm5571sup1.cif

Supporting information file. DOI: 10.1107/S2056989020009779/wm5571Isup3.cdx

Structure factors: contains datablock(s) I. DOI: 10.1107/S2056989020009779/wm5571Isup4.hkl

CCDC reference: 2017141

Additional supporting information:  crystallographic information; 3D view; checkCIF report

## Figures and Tables

**Table 1:** Hydrogen-bond geometry (Å, °)

*D*—H⋯*A*	*D*—H	H⋯*A*	*D*⋯*A*	*D*—H⋯*A*
O4—H4*A*⋯O2^i^	0.85	1.97	2.818 (4)	180
O4—H4*B*⋯O3^ii^	0.85	2.04	2.821 (4)	152
C4—H4⋯O1^iii^	0.93	2.40	3.217 (4)	146
C5—H5⋯O1^iv^	0.93	2.40	3.321 (4)	170
C6—H6⋯O4	0.93	2.39	3.209 (4)	147

Symmetry codes: (i) 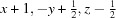; (ii) 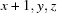; (iii) 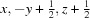; (iv) 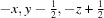.

**Table 2:** Experimental details

Crystal data	
Chemical formula	C_16_H_26_N_4_O_6_S_2_·2H_2_O
*M*_r_	470.56
Crystal system, space group	Monoclinic, *P*2_1_/*c*
Temperature (K)	300
*a*, *b*, *c* (Å)	5.6085 (4), 18.1641 (11), 10.6884 (7)
β (°)	97.860 (4)
*V* (Å^3^)	1078.63 (12)
*Z*	2
Radiation type	Cu *K*α
μ (mm^−1^)	2.69
Crystal size (mm)	0.20 × 0.12 × 0.07

Data collection	
Diffractometer	Bruker D8 VENTURE
Absorption correction	Multi-scan (*SADABS*; Krause *et al.*, 2015 ▸)
*T*_min_, *T*_max_	0.702, 0.820
No. of measured, independent and observed [*I* > 2σ(*I*)] reflections	8276, 1841, 1483
*R*_int_	0.052
(sin θ/λ)_max_ (Å^−1^)	0.596

Refinement	
*R*[*F* ^2^ > 2σ(*F* ^2^)], *wR*(*F* ^2^), *S*	0.048, 0.109, 1.11
No. of reflections	1841
No. of parameters	136
H-atom treatment	H-atom parameters constrained
Δρ_max_, Δρ_min_ (e Å^−3^)	0.40, −0.29

Computer programs: *APEX3* and *SAINT* (Bruker 2017 ▸), *SHELXT* (Sheldrick, 2015*a* ▸), *SHELXL* (Sheldrick, 2015*b* ▸), *Mercury* (Macrae *et al.*, 2020 ▸), *WinGX* (Farrugia, 2012 ▸), *OLEX2* (Dolomanov *et al.*, 2009 ▸) and *publCIF* (Westrip, 2010 ▸).

## supplementary crystallographic information

### Crystal data

**Table:**

C_16_H_26_N_4_O_6_S_2_·2H_2_O	*F*(000) = 500
*M**_r_* = 470.56	*D*_x_ = 1.449 Mg m^−^^3^
Monoclinic, *P*2_1_/*c*	Cu *K*α radiation, λ = 1.54178 Å
*a* = 5.6085 (4) Å	Cell parameters from 8513 reflections
*b* = 18.1641 (11) Å	θ = 4.8–70.0°
*c* = 10.6884 (7) Å	µ = 2.69 mm^−^^1^
β = 97.860 (4)°	*T* = 300 K
*V* = 1078.63 (12) Å^3^	Prism, colourless
*Z* = 2	0.20 × 0.12 × 0.07 mm

### Data collection

**Table:**

Bruker D8 VENTURE diffractometer	1483 reflections with *I* > 2σ(*I*)
Radiation source: microfocus sealed tube	*R*_int_ = 0.052
ω and φ scans	θ_max_ = 66.8°, θ_min_ = 4.8°
Absorption correction: multi-scan (*SADABS*; Krause *et al.*, 2015)	*h* = −6→6
*T*_min_ = 0.702, *T*_max_ = 0.820	*k* = −21→21
8276 measured reflections	*l* = −12→11
1841 independent reflections	

### Refinement

**Table:**

Refinement on *F*^2^	Primary atom site location: structure-invariant direct methods
Least-squares matrix: full	Secondary atom site location: structure-invariant direct methods
*R*[*F*^2^ > 2σ(*F*^2^)] = 0.048	Hydrogen site location: mixed
*wR*(*F*^2^) = 0.109	H-atom parameters constrained
*S* = 1.11	*w* = 1/[σ^2^(*F*_o_^2^) + (0.0303*P*)^2^ + 0.8877*P*] where *P* = (*F*_o_^2^ + 2*F*_c_^2^)/3
1841 reflections	(Δ/σ)_max_ < 0.001
136 parameters	Δρ_max_ = 0.40 e Å^−^^3^
0 restraints	Δρ_min_ = −0.29 e Å^−^^3^

### Special details

**Table:**

Geometry. All esds (except the esd in the dihedral angle between two l.s. planes) are estimated using the full covariance matrix. The cell esds are taken into account individually in the estimation of esds in distances, angles and torsion angles; correlations between esds in cell parameters are only used when they are defined by crystal symmetry. An approximate (isotropic) treatment of cell esds is used for estimating esds involving l.s. planes.

### Fractional atomic coordinates and isotropic or equivalent isotropic displacement parameters (Å^2^)

**Table:**

	*x*	*y*	*z*	*U*_iso_*/*U*_eq_	
S1	−0.01051 (13)	0.32408 (4)	0.27909 (7)	0.0384 (2)	
O1	0.0178 (4)	0.36082 (12)	0.1620 (2)	0.0515 (6)	
O2	−0.0556 (5)	0.37554 (13)	0.3769 (2)	0.0684 (7)	
O3	−0.1853 (4)	0.26460 (13)	0.2622 (2)	0.0605 (7)	
N1	0.4379 (4)	0.12377 (12)	0.4186 (2)	0.0342 (5)	
N2	0.4693 (4)	0.03758 (12)	0.2835 (2)	0.0397 (6)	
C1	0.2680 (5)	0.28051 (15)	0.3317 (3)	0.0387 (7)	
H1A	0.295761	0.242246	0.272092	0.046*	
H1B	0.396416	0.316390	0.333023	0.046*	
C2	0.2778 (5)	0.24673 (15)	0.4622 (3)	0.0407 (7)	
H2A	0.126464	0.222028	0.468117	0.049*	
H2B	0.296498	0.285719	0.524741	0.049*	
C3	0.4824 (6)	0.19192 (15)	0.4923 (3)	0.0414 (7)	
H3A	0.631092	0.214116	0.473993	0.050*	
H3B	0.501309	0.180196	0.581604	0.050*	
C4	0.2637 (5)	0.07226 (16)	0.4320 (3)	0.0442 (8)	
H4	0.152627	0.074210	0.489045	0.053*	
C5	0.2834 (6)	0.01861 (16)	0.3477 (3)	0.0470 (8)	
H5	0.188704	−0.023476	0.335243	0.056*	
C6	0.5586 (5)	0.10126 (15)	0.3272 (3)	0.0383 (7)	
H6	0.685369	0.126268	0.298589	0.046*	
C7	0.5562 (6)	−0.00487 (17)	0.1802 (3)	0.0516 (8)	
H7A	0.517965	−0.056495	0.189484	0.062*	
H7B	0.729934	−0.000435	0.187416	0.062*	
C8	0.4471 (6)	0.02087 (17)	0.0518 (3)	0.0462 (8)	
H8A	0.274204	0.013577	0.041893	0.055*	
H8B	0.477584	0.073117	0.043983	0.055*	
O4	0.9450 (6)	0.13610 (16)	0.1398 (3)	0.0982 (11)	
H4A	0.944211	0.132449	0.060413	0.147*	
H4B	0.898201	0.179850	0.150683	0.147*	

### Atomic displacement parameters (Å^2^)

**Table:**

	*U*^11^	*U*^22^	*U*^33^	*U*^12^	*U*^13^	*U*^23^
S1	0.0413 (4)	0.0408 (4)	0.0350 (4)	0.0084 (3)	0.0128 (3)	0.0048 (3)
O1	0.0632 (15)	0.0539 (13)	0.0404 (12)	0.0105 (11)	0.0174 (11)	0.0153 (10)
O2	0.097 (2)	0.0648 (15)	0.0463 (14)	0.0381 (14)	0.0195 (13)	−0.0033 (11)
O3	0.0407 (13)	0.0686 (15)	0.0714 (17)	−0.0097 (11)	0.0046 (12)	0.0164 (12)
N1	0.0408 (13)	0.0316 (12)	0.0313 (13)	0.0045 (10)	0.0087 (11)	−0.0006 (10)
N2	0.0527 (16)	0.0335 (13)	0.0344 (14)	0.0073 (11)	0.0114 (12)	−0.0036 (10)
C1	0.0362 (16)	0.0368 (15)	0.0447 (18)	0.0024 (12)	0.0116 (14)	0.0029 (13)
C2	0.0528 (19)	0.0371 (15)	0.0332 (16)	0.0063 (13)	0.0095 (14)	−0.0037 (12)
C3	0.0488 (18)	0.0397 (16)	0.0340 (16)	0.0047 (13)	−0.0008 (14)	−0.0071 (13)
C4	0.0486 (19)	0.0485 (17)	0.0389 (18)	−0.0005 (14)	0.0180 (15)	0.0016 (14)
C5	0.056 (2)	0.0391 (17)	0.0474 (19)	−0.0063 (14)	0.0138 (16)	−0.0014 (14)
C6	0.0431 (17)	0.0391 (16)	0.0348 (16)	0.0026 (13)	0.0126 (14)	0.0028 (13)
C7	0.072 (2)	0.0434 (17)	0.0413 (18)	0.0170 (16)	0.0155 (17)	−0.0081 (14)
C8	0.056 (2)	0.0445 (17)	0.0388 (18)	0.0079 (15)	0.0103 (15)	−0.0083 (14)
O4	0.154 (3)	0.082 (2)	0.0673 (19)	−0.0355 (19)	0.047 (2)	−0.0023 (15)

### Geometric parameters (Å, º)

**Table:**

S1—O1	1.446 (2)	C2—H2B	0.9700
S1—O2	1.450 (2)	C3—H3A	0.9700
S1—O3	1.453 (2)	C3—H3B	0.9700
S1—C1	1.773 (3)	C4—C5	1.342 (4)
N1—C6	1.327 (3)	C4—H4	0.9300
N1—C4	1.374 (4)	C5—H5	0.9300
N1—C3	1.470 (3)	C6—H6	0.9300
N2—C6	1.320 (4)	C7—C8	1.499 (4)
N2—C5	1.368 (4)	C7—H7A	0.9700
N2—C7	1.482 (3)	C7—H7B	0.9700
C1—C2	1.517 (4)	C8—C8^i^	1.528 (5)
C1—H1A	0.9700	C8—H8A	0.9700
C1—H1B	0.9700	C8—H8B	0.9700
C2—C3	1.520 (4)	O4—H4A	0.8500
C2—H2A	0.9700	O4—H4B	0.8499
			
O1—S1—O2	112.16 (14)	C2—C3—H3A	109.3
O1—S1—O3	112.80 (14)	N1—C3—H3B	109.3
O2—S1—O3	112.80 (15)	C2—C3—H3B	109.3
O1—S1—C1	106.52 (13)	H3A—C3—H3B	107.9
O2—S1—C1	106.97 (15)	C5—C4—N1	107.4 (2)
O3—S1—C1	104.93 (13)	C5—C4—H4	126.3
C6—N1—C4	108.0 (2)	N1—C4—H4	126.3
C6—N1—C3	126.0 (2)	C4—C5—N2	107.0 (3)
C4—N1—C3	126.1 (2)	C4—C5—H5	126.5
C6—N2—C5	108.7 (2)	N2—C5—H5	126.5
C6—N2—C7	124.9 (2)	N2—C6—N1	108.9 (2)
C5—N2—C7	126.4 (3)	N2—C6—H6	125.6
C2—C1—S1	113.08 (19)	N1—C6—H6	125.6
C2—C1—H1A	109.0	N2—C7—C8	112.6 (2)
S1—C1—H1A	109.0	N2—C7—H7A	109.1
C2—C1—H1B	109.0	C8—C7—H7A	109.1
S1—C1—H1B	109.0	N2—C7—H7B	109.1
H1A—C1—H1B	107.8	C8—C7—H7B	109.1
C1—C2—C3	113.0 (2)	H7A—C7—H7B	107.8
C1—C2—H2A	109.0	C7—C8—C8^i^	111.0 (3)
C3—C2—H2A	109.0	C7—C8—H8A	109.5
C1—C2—H2B	109.0	C8^i^—C8—H8A	109.5
C3—C2—H2B	109.0	C7—C8—H8B	109.2
H2A—C2—H2B	107.8	C8^i^—C8—H8B	109.4
N1—C3—C2	111.7 (2)	H8A—C8—H8B	108.0
N1—C3—H3A	109.3	H4A—O4—H4B	104.5
			
O1—S1—C1—C2	173.2 (2)	C6—N2—C5—C4	−0.4 (4)
O2—S1—C1—C2	53.1 (2)	C7—N2—C5—C4	−179.5 (3)
O3—S1—C1—C2	−66.9 (2)	C5—N2—C6—N1	0.6 (3)
S1—C1—C2—C3	163.8 (2)	C7—N2—C6—N1	179.7 (2)
C6—N1—C3—C2	111.4 (3)	C4—N1—C6—N2	−0.6 (3)
C4—N1—C3—C2	−68.4 (4)	C3—N1—C6—N2	179.6 (2)
C1—C2—C3—N1	−71.0 (3)	C6—N2—C7—C8	−83.8 (4)
C6—N1—C4—C5	0.3 (3)	C5—N2—C7—C8	95.1 (4)
C3—N1—C4—C5	−179.9 (3)	N2—C7—C8—C8^i^	176.4 (3)
N1—C4—C5—N2	0.1 (4)		

Symmetry code: (i) −*x*+1, −*y*, −*z*.

### Hydrogen-bond geometry (Å, º)

**Table:**

*D*—H···*A*	*D*—H	H···*A*	*D*···*A*	*D*—H···*A*
O4—H4*A*···O2^ii^	0.85	1.97	2.818 (4)	180
O4—H4*B*···O3^iii^	0.85	2.04	2.821 (4)	152
C4—H4···O1^iv^	0.93	2.40	3.217 (4)	146
C5—H5···O1^v^	0.93	2.40	3.321 (4)	170
C6—H6···O4	0.93	2.39	3.209 (4)	147

Symmetry codes: (ii) *x*+1, −*y*+1/2, *z*−1/2; (iii) *x*+1, *y*, *z*; (iv) *x*, −*y*+1/2, *z*+1/2; (v) −*x*, *y*−1/2, −*z*+1/2.

## References

[bb1] Asensio, J. M., Gómez-Sal, P., Andrés, R. & de Jesús, E. (2017). *Dalton Trans.* **46**, 6785–6797. 10.1039/c7dt00643h 28492647

[bb2] Bruker (2017). *APEX3* and *SAINT*. Bruker AXS Inc., Madison, Wisconsin, USA, 2017.

[bb3] De, S., Udvardy, A., Czégéni, C. E. & Joó, F. (2019). *Coord. Chem. Rev.* **400**, 213038.

[bb4] Dolomanov, O. V., Bourhis, L. J., Gildea, R. J., Howard, J. A. K. & Puschmann, H. (2009). *J. Appl. Cryst.* **42**, 339–341.

[bb5] Farrugia, L. J. (2012). *J. Appl. Cryst.* **45**, 849–854.

[bb6] Groom, C. R., Bruno, I. J., Lightfoot, M. P. & Ward, S. C. (2016). *Acta Cryst.* B**72**, 171–179. 10.1107/S2052520616003954 PMC4822653 27048719

[bb7] Hung, F. F., To, W. P., Zhang, J. J., Ma, C., Wong, W. Y. & Che, C. M. (2014). *Chem. Eur. J.* **20**, 8604–8614. 10.1002/chem.201403103 24957269

[bb8] Kohmoto, S., Okuyama, S., Yokota, N., Takahashi, M., Kishikawa, K., Masu, H. & Azumaya, I. (2012). *J. Mol. Struct.* **1015**, 6–11.

[bb9] Krause, L., Herbst-Irmer, R., Sheldrick, G. M. & Stalke, D. (2015). *J. Appl. Cryst.* **48**, 3–10. 10.1107/S1600576714022985 PMC4453166 26089746

[bb10] Liu, H. F., Zeng, F.-X., Deng, L., Liao, B., Pang, H. & Guo, Q. X. (2013). *Green Chem.* **15**, 81–84.

[bb11] Macrae, C. F., Sovago, I., Cottrell, S. J., Galek, P. T. A., McCabe, P., Pidcock, E., Platings, M., Shields, G. P., Stevens, J. S., Towler, M. & Wood, P. A. (2020). *J. Appl. Cryst.* **53**, 226–235. 10.1107/S1600576719014092 PMC6998782 32047413

[bb12] Papini, G., Pellei, M., Lobbia, G. G., Burini, A. & Santini, G. (2009). *Dalton Trans.* pp. 6985–6990. 10.1039/b906994a 20449140

[bb13] Sheldrick, G. M. (2015*a*). *Acta Cryst.* A**71**, 3–8.

[bb14] Sheldrick, G. M. (2015*b*). *Acta Cryst.* C**71**, 3–8.

[bb15] Westrip, S. P. (2010). *J. Appl. Cryst.* **43**, 920–925.

[bb16] Xu, Y., Liang, J., Ren, X., Jiang, M., Wei, P. & Ouyang, P. (2012). *Nanjing Gongye Daxue Xuebao, Ziran Kexueban* **34**, 48–52.

[bb17] Zeng, F. X., Liu, H. F., Deng, L., Liao, B., Pang, H. & Guo, Q. X. (2013). *ChemSusChem*, **6**, 600–603. 10.1002/cssc.201200841 23468313

